# Clinical Notes on Wound Dressing

**Published:** 1906-01

**Authors:** Charles N. Cook

**Affiliations:** Philadelphia, Pa.


### Clinical Notes on Wound Dressing.

By CHARLES N. COOK, M. D., Philadelphia, Pa.

When the practitioner seeks an ideal surgical dressing, there are two main points to be borne in mind. First, that it must maintain asepsis and eliminate infection; and second, that it must assist and not retard nature in her effort to repair the tissue.

Many of the germicides and antiseptics when carefully considered, will be weighed in the balance and found wanting, and to the unprejudiced mind seem to have been selected without regard to any other consideration than their germicidal power. It is a fact that solutions of corrisive sublimate destroy bacterial life; it is equally certain that they coagulate serous albumen. This is the case also when carbolic acid, formalin, and other preparations of that class are used, and thus we see while it is true they destroy germ life, they also retard nature's effort in making repair. In the writer's opinion the ideal surgical dressing should conform to the following requirements:

I.—It should be in harmony with the fluids of the tissues; i. e., it should be distinctly alkaline in reaction and of apropriate specific gravity to the liquor sanguine.

II.—It should be capable of promoting rapid exosmosis. This is most important for by this natural phenomenon the toxins secreted by the microorganisms are prevented from being taken up by the circulation and at the same time controlling and eliminating sepsis. The property of producing exosmosis renders the antiseptic antiphlogistic a most valuable feature.

III.—It should not coagulate serous albumen or the fibrins of the tissues.

IV.—It should possess deodorant qualities.

V.—While not absolutely essential it is highly desirable that it should be non-toxic in character. Such a preparation does exist and all that any surgeon has to do in order to convince himself is to make a practical trial of it.

I feel confident that he will agree that we have at hand an antiseptic surgical dressing which conforms to the requirements which I have stated above and which is manifestly superior to solutions of bichloride of mercury, carbolic acid, formaldehyde, lysol creolin, and other similar agents.

I refer to the well known glycothymoline. I have made a careful trial of it in a great many cases and in every case it has afforded almost perfect results.

Below I cite two cases taken at random from my notes, which will illustrate.

Case I.—Willie R., age 5 years, while at play fell on stone steps, cutting a gash five inches and a half long in the forehead just over the right eye. I put in five stitches and dressed with pure glycothymoline. Nothing could have done better; no pus, hardly any scar resulted.

Case II.—Man employed in factory had hand caught in machine which took a slice off the under finger of right hand, cutting it from base to tip. I wrapped it in gauze saturated with pure glycothymoline. This dressing was kept moistened in the glycothymoline and not disturbed for over a week when I redressed it in the same manner. In two weeks the man was back again at work.

In infected wounds I have found it all that can be desired. If there be any one who is in doubt, let him try this ideal surgical dressing, glycothymoline, and I am sure he will become convinced.

1637 N. Twenty-second Street.

